# Ti_2_NiCoSnSb - a new half-Heusler type high-entropy alloy showing simultaneous increase in Seebeck coefficient and electrical conductivity for thermoelectric applications

**DOI:** 10.1038/s41598-019-41818-6

**Published:** 2019-03-29

**Authors:** Anirudha Karati, M. Nagini, Sanyukta Ghosh, Rajashekhara Shabadi, K. G. Pradeep, Ramesh Chandra Mallik, B. S. Murty, U. V. Varadaraju

**Affiliations:** 1 Department of Chemistry, Indian Institute of Technology Madras, Chennai, India; 2 Department of Metallurgical and Materials Engineering, Indian Institute of Technology Madras, Chennai, India; 3 Thermoelectric Materials and Devices Laboratory, Department of Physics, Indian Institute of Science, Bangalore, India; 4 Faculty of Science and Technology, UMET, University of Lille, Villeneuve-d’Ascq, France

## Abstract

A new single phase high entropy alloy, Ti_2_NiCoSnSb with half-Heusler (HH) structure is synthesized for the first time by vacuum arc melting (VAM) followed by ball-milling (BM). The BM step is necessary to obtain the single phase. Local electrode atom probe (LEAP) analysis showed that the elements are homogeneously and randomly distributed in the HH phase without any clustering tendency. When the BM was carried out for 1 hour on the VAM alloy, microcrystalline alloy is obtained with traces of Sn as secondary phase. When BM was carried out for 5 h, single HH phase formation is realized in nanocrystalline form. However, when the BM samples were subjected to Spark plasma sintering (SPS), secondary phases were formed by the decomposition of primary phase. Nanostructuring leads to simultaneous increase in S and σ with increasing temperature. The micro (1 h BM-SPS) and nanocrystalline (5 h BM-SPS) alloys exhibited a power factor (S^2^σ) of 0.57 and 1.02 mWm^−1^K^−2^, respectively, at 860 K. The microcrystalline sample had a total thermal conductivity similar to bulk TiNiSn sample. The nanocrystalline alloy exhibited a ZT of 0.047 at 860 K. The microcrystalline alloy showed a ZT to 0.144 at 860 K, in comparison to the nanocrystalline alloy.

## Introduction

HH alloys are ternary intermetallic compounds with three interpenetrating sub-lattices. They crystallize in MgAgAs crystal structure with F-43m symmetry. The HH alloys having a valence electron count of 18 are found to be semi-conducting in nature. Compounds such as TiNiSn and TiCoSb are some of the well-studied HH alloys for thermoelectric (TE) applications. They have a narrow band gap and have good mechanical and thermal properties over other TE systems. HH alloys exhibit high Seebeck coefficient and electrical conductivity. However, they possess a very high thermal conductivity which serves as a bottleneck in achieving high TE figure of merit (ZT) in these materials^1–3^. Recent studies have thus been primarily devoted to decreasing thermal conductivity in these alloys by nanostructuring^4^, introduction of *in-situ* and *ex-situ* secondary phases^5^ and solid solution approach to create point defects^6^ to name a few.

HH alloys have been synthesized by vacuum arc melting (VAM)^7^, induction melting^8^, solid state synthesis^9^ and mechanical alloying (MA) followed by SPS (MA-SPS)^10^. However, in most of these cases, long annealing time is required to obtain single phase alloys^11^. In some cases, secondary phases cannot be eliminated even after long time annealing^12^. Long time annealing has also proven to degrade TE properties in TiCoSb owing to the loss of Sb or introduction of structural disorder^13^.

Contrary to the traditional methods of alloying, a new strategy that employs the usage of multi-principal elements in equiatomic or near equiatomic compositions has come into prominence in the last decade. These alloys were discovered almost simultaneously by Cantor *et al*.^14^ and Yeh *et al*.^15^. The latter coined the term “*High-Entropy Alloys*” owing to their high configurational entropy in comparison to traditional alloys. Most of these alloys end up having simple solid solution structures such as BCC, FCC and HCP instead of intermetallic compounds owing to the high mixing entropy in these systems^16^. They exhibit superior mechanical properties^17^, grain growth resistance at high temperature^18^, high oxidation and corrosion resistance^19,20^. HEAs have also found their use in functional applications and have exhibited good magnetic, magnetocaloric, superconducting and hydrogen storage properties^21^. Additionally, HEAs have also been used for TE applications^21^. A TE material converts heat to electricity and vice versa. The efficacy of conversion is defined by the figure of merit Z = S^2^σ/κ where, S is the thermopower or Seebeck coefficient, σ is the electrical conductivity and κ is the total thermal conductivity which comprises of two components namely, the electronic contribution (κ_e_) and the lattice contribution (κ_L_). The first report of HEA as TE material was by Shafeie *et al*. in AlCoCrFeNi^22^. However, whilst it had high electrical conductivity, it exhibited low Seebeck coefficient and high thermal conductivity and in turn low ZT. Among the HEAs exhibiting thermoelectric properties, (BiSbSe_1.5_Te_1.5_)_1−x_Ag_x_^23^, (PbSnSeTe)_1−x_La_x_^24^ and Nb_0.2_(TiVHfMoZr)_0.8_FeSb^25^ designated as HE materials by the authors, exhibited high ZT values of 0.63 at 450 K and 0.8 at 873 K and 0.88 at 873 K, respectively.

In the present work, we report the synthesis of a new HH-type HEA Ti_2_NiCoSnSb for the first time. Micro- and nanocrystalline microstructures were obtained by ball milling for 1 and 5 h, respectively. In the manuscript they will be referred to as microcrystalline and nanocrystalline alloys, respectively. The alloy powder was consolidated by SPS and the TE properties were measured. The microcrystalline alloy exhibits higher ZT value than the nanocrystalline alloy.

## Results

### Synthesis of Ti_2_NiCoSnSb microcrystalline alloy by VAM

**Figure 1:**
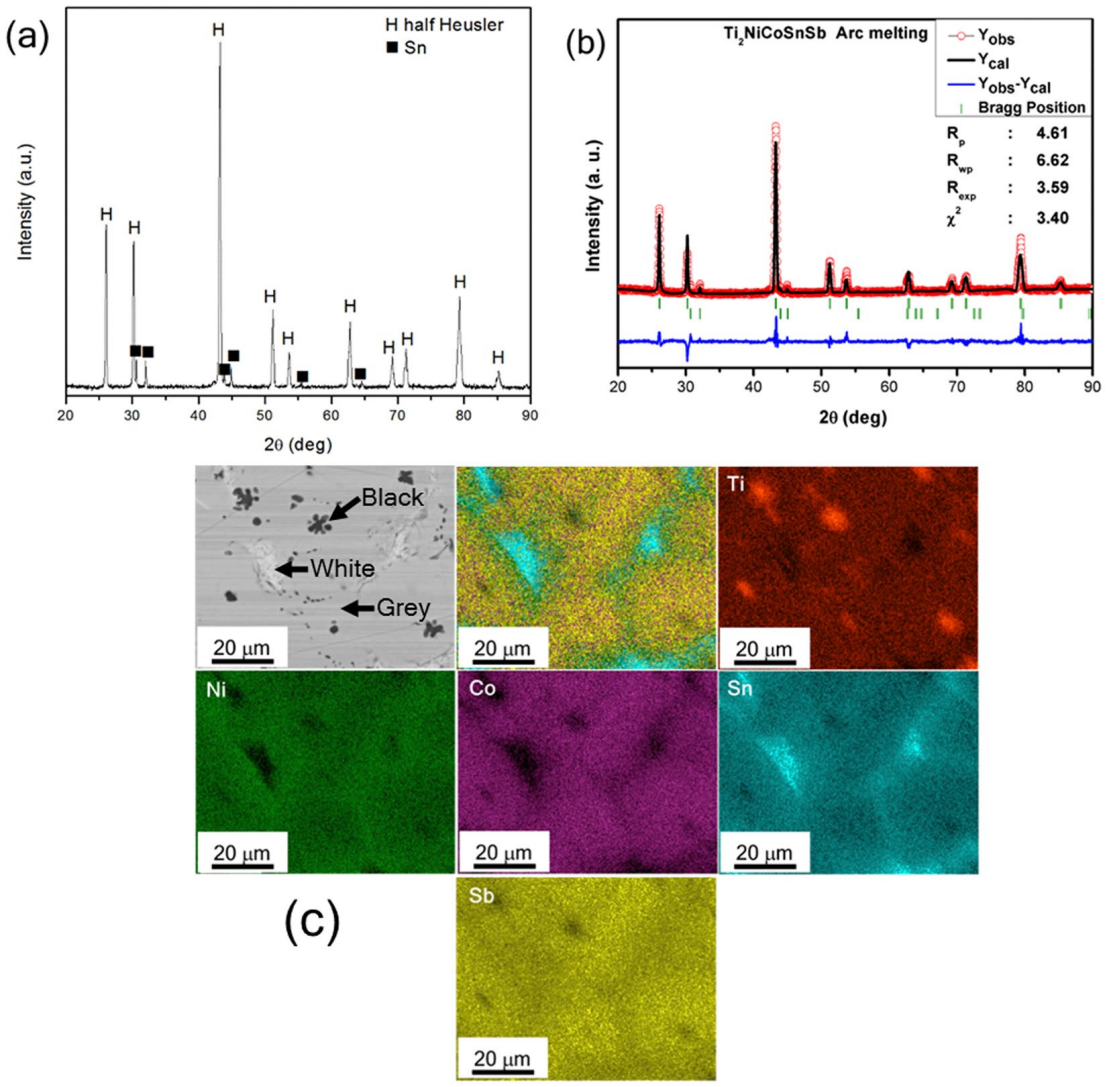
(**a**) XRD pattern, (**b**) Rietveld pattern of the VAM Ti_2_NiCoSnSb alloy and (**c**) EDS Elemental mapping of the cast alloy confirming the presence of 3 phases.

The XRD pattern of VAM Ti_2_NiCoSnSb alloy showed HH phase along with small peaks of Sn (Fig. 1a). The Rietveld refinement pattern (Fig. 1b) confirmed the HH phase formation crystallizing in the *F* $\bar{4}$*3m* symmetry. The fraction of Sn phase was calculated through Relative Intensity Ratio (RIR) analysis^26^ to be 8%. The (020) peak of Sn phase and (220) peak of HH phase for analysis. SEM imaging in backscattered electron (BSE) mode revealed three phases in the microstructure as shown in Fig. 1c. Elemental mapping was carried out for the alloy (Fig. 1c) and it confirmed the three regions to be having different compositions. The image analysis on the BSE micrographs confirmed the white region to have a volume fraction of 7% whilst the black region was found to have a volume fraction of 3% as shown in Table 1. The white and black regions were found to be rich in Sn and Ti, respectively. The grey region was found to have a uniform distribution of all the elements present and corresponds to the majority phase namely, the HH phase. The quantitative chemical composition analysis of the three regions in the cast alloy is shown in Table S1 and agrees well with the distributions observed in the elemental mapping. A minor loss in Sn and a slight excess in Ti were observed in the overall composition. The concentrations of the other elements are in accordance with the nominal composition. Elemental mapping was carried out on the grey region (Fig. S1), which did not reveal segregation of any element in the region.

**Table 1:** Lattice parameter (N = 3) and volume of unit cell (N = 3) of the half-Heusler alloy using Nelson-Riley parameter and volume fraction analysis carried out by RIR method (XRD analysis) and from SEM (N = 3).

Alloy	Lattice parameter of HH phase (nm)	Volume of unit cell of HH phase (nm^3^)	Volume fraction of HH phase		Volume fraction of Sn		Volume fraction of Ti		Volume fraction of TiC		Volume fraction of Ni_3_Sn_4_	
			XRD	SEM	XRD	SEM	XRD	SEM	XRD	SEM	XRD	SEM
TiNiSn^32^	0.5921	0.2076	1	1	0	0	0	0	0	0	0	0
As-cast Ti_2_NiCo SnSb	0.5915 ± 0.0022	0.2070 ± 0.0002	0.92	0.90 ± 0.004	0.08	0.07 ± 0.006	0	0.03 ± 0.002	0	0	0	0
TiCoSb^31^	0.5882	0.2035	1	1	0	0	0	0	0	0	0	0
1 h BM	0.5917 ± 0.0003	0.2071 ± 0.0002	0.96	—	0.04	0	0	0	0	0	0	0
5 h BM	0.5921 ± 0.0009	0.2076 ± 0.0001	1	—	0	0	0	0	0	0	0	0
1 h BM-SPS	0.5904 ± 0.0014	0.2058 ± 0.0001	0.95	0.94 ± 0.001	0	0	0	0	0.05	0.05 ± 0.001	0	0.007 ± 0.001
5 h BM-SPS	0.5901 ± 0.0029	0.2054 ± 0.0003	0.89	0.88 ± 0.003	0	0	0	0	0.11	0.11 ± 0.002	0	0.011 ± 0.001

Each composition in the table is the average value of 3 different measurement points.

**Figure 2:**
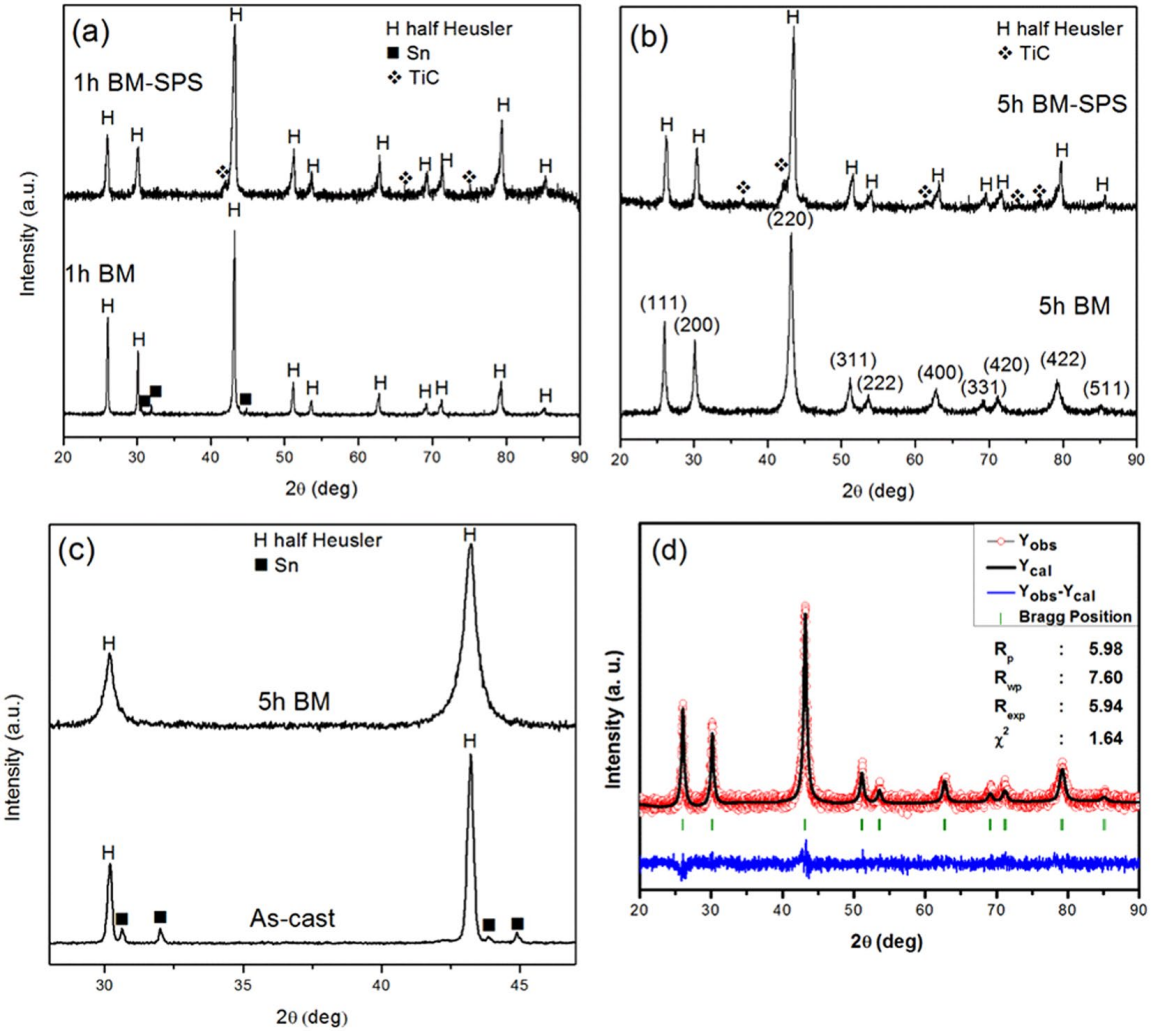
XRD pattern of (**a**) 1 h BM and 1 h BM-SPS alloy, (**b**) 5 h BM and 5 h BM-SPS alloy, (**c**) zoomed in region of 5 h BM alloy showing absence of Sn peaks, (**d**) Rietveld refinement pattern of the 5 h BM alloy.

The cast alloy was found to be very brittle and further machining of it to make samples for TE property measurements was difficult. Hence, the cast alloy was hand crushed and ball milled for 1 h. The milled powder showed HH phase (Fig. 2a) with a minor phase (4%) of Sn still remaining. The milled powder upon sintering showed absence of Sn peaks. However, the formation of TiC phase was observed after sintering (Fig. 2a). The microstructure of the sintered pellet is shown in Fig. S2. The low magnification image in Fig. S2a displays 3 regions namely, grey, white and black. The presence of white and black regions are further confirmed from the high magnification micrograph as shown in Fig. S2b. The white region was found to be rich in Ni and Sn whilst the black region was rich in Ti and C. The volume fraction of the black and white regions in the 1 h BM-SPS samples are found to be 5% and 1%, respectively as indicated in Table 1. The quantitative data on the overall composition of the sample and of the individual regions in the BM-SPS alloys is shown in Table S2.

### Synthesis of Ti_2_NiCoSnSb nanocrystalline alloy by ball milling followed by SPS

**Figure 3:**
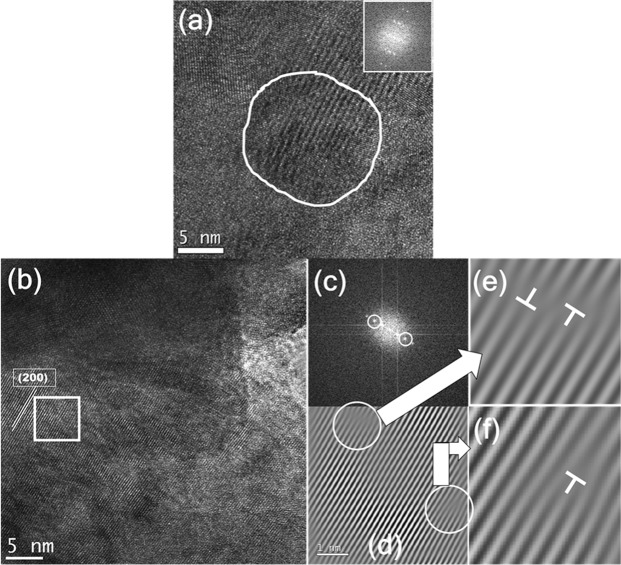
(**a**) HR-TEM image showing a nanocrystallite in 5 h BM alloy. The inset confirms the single crystal nature of the crystallite, (**b**) (200) plane observed in 5 h BM alloy, (**c**) The FFT from the region selected in (**b**) showing the SADP from the region, (**d**) The inverse FFT image from the selected points in (**c**) showing lattice fringes, (**e**,**f**) dislocations observed in the lattice corresponding to the regions indicated in (**d**).

The cast alloy was subjected to ball milling for 5 h to make it nanocrystalline. The peaks in the XRD pattern matched with those corresponding to the HH structure as shown in Fig. 2b. No secondary phase is seen. The XRD pattern of the sintered pellet exhibited the formation of TiC in addition to the presence of the HH phase. Furthermore, it was observed that the peaks corresponding to Sn phase were absent as observed in the enlarged XRD pattern in Fig. 2c. The Rietveld refinement (Fig. 2d) of the 5 h BM pattern confirmed the presence of a single phase in the alloy. In order to validate the dissolution of Sn in the alloy, elemental mapping was carried out on the 5 h BM sample as shown in Fig. S3. The alloy showed absence of any elemental segregation especially for Co, Ni, Sn and Sb. A few regions exhibiting Ti segregation were however observed. Figure 3a shows the presence of nanocrystalline grains in the 5 h BM alloy. The average size of the crystallites is found to be ~12 nm from TEM analysis which is in accordance with the data obtained from XRD (using Williamson-Hall analysis). The HH phase was confirmed from the indexing of the (200) plane as shown in Fig. 3b. The SAED pattern that was generated by taking the fast Fourier transformation (FFT) from the region selected in Fig. 3b is displayed in Fig. 3c. The inverse fast Fourier transformation (I-FFT) was carried out on Fig. 3c and is displayed in Fig. 3d. The images in Fig. 3e,f show zoomed in regions from Fig. 3d showing the presence of dislocations in the matrix phase.

The microstructure of 5 h BM alloy sintered at 1173 K is shown in Fig. S4. The microstructure comprises of 3 regions, namely, grey, white and black. The black regions are Ti-rich regions. This could be attributed to the TiC that was observed in the XRD pattern in Fig. 2b. The volume fraction of TiC is higher in the 5 h BM-SPS sample (11%) in comparison to the 1 h BM-SPS sample (5%) as shown in Table 1. The grey region showed a composition that was very similar to the overall composition of the sample. The white region was a Ni/Sn rich system which could be attributed to Ni_3_Sn_4_. The difference in volume fraction of the white region is however, not large between 1 h BM-SPS (0.7%) and 5 h BM-SPS samples (1.1%). The plausible mechanism for this phase segregation reaction can be:

$${{\rm{Ti}}}_{{\rm{2}}}{\rm{NiCoSnSb}}+{\rm{x}}\,{\rm{C}}\to {{\rm{Ti}}}_{2-x}{{\rm{Ni}}}_{1-y}{{\rm{CoSn}}}_{{\rm{1}}-{\rm{y}}}{\rm{Sb}}+{\rm{x}}\,{\rm{TiC}}+{\rm{y}}\,{\rm{Ni}} \mbox{-} {\rm{Sn}}$$

Carbon pick up from toluene occurs during the ball milling process. This leads to the formation of TiC after SPS. Due to unavailability of adequate Ti for the HH phase to be stable, certain fraction of Ni_3_Sn_4_ gets precipitated out. This can be further confirmed by the observation of white regions (Ni_3_Sn_4_) near the black regions (TiC) in the microstructure.

### Electronic and thermal transport properties

**Figure 4:**
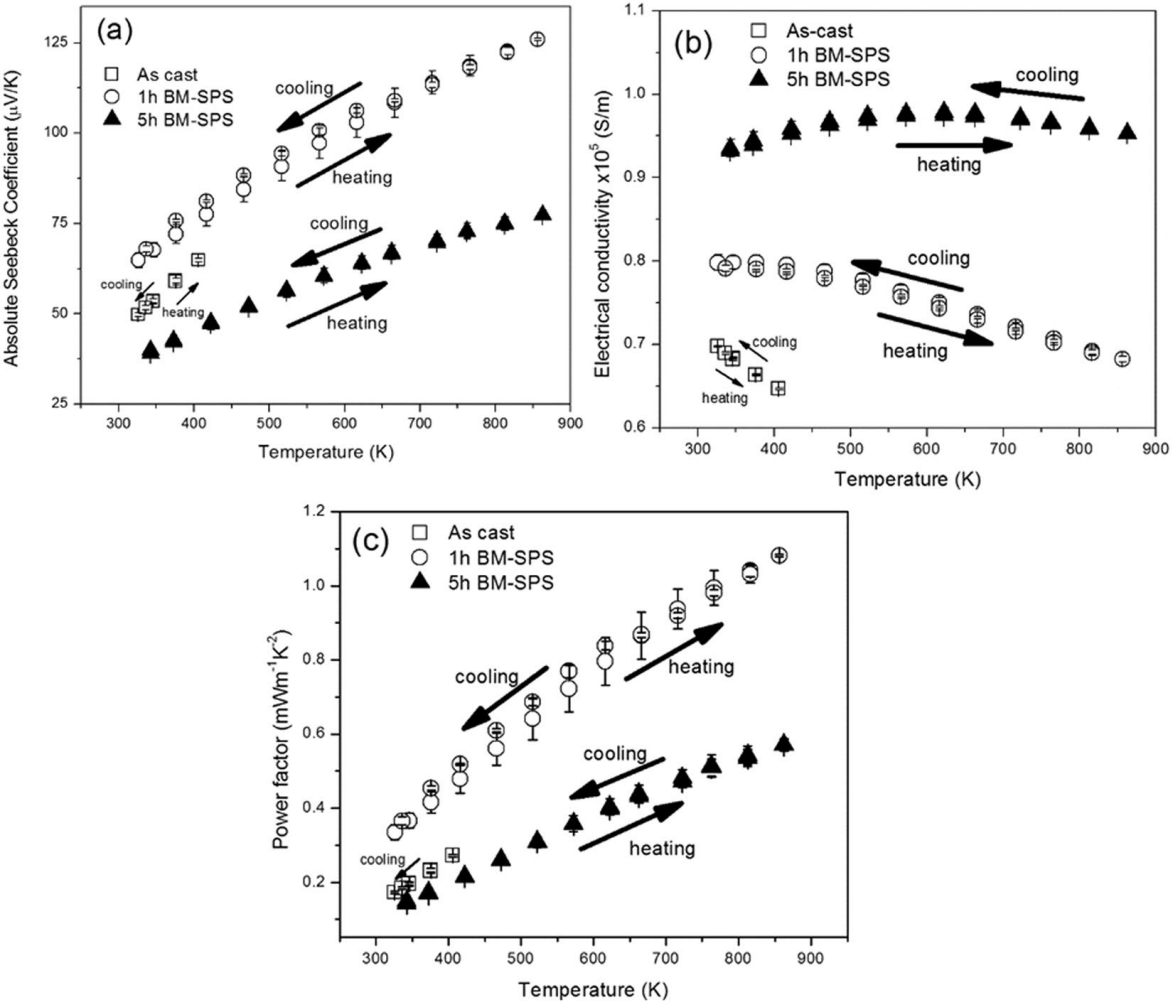
Variation of (**a**) Seebeck coefficient, (**b**) electrical conductivity and (**c**) power factor as a function of temperature for as-cast, 1 h BM-SPS and 5 h BM-SPS samples (heating and cooling cycles). Each data point is an average of 3 measurements. The error bars are indicated.

The Seebeck coefficient (S) and electrical conductivity (σ) as a function of temperature are shown in Fig. 4a,b, respectively. For a TE material, the S and σ are inversely proportional to the carrier concentration. Hence, an increase in S is generally coupled with a decrease in σ and vice versa. The microcrystalline alloys (as-cast and 1 h BM-SPS) show an increase in S and a decrease in σ with increasing temperature. On the other hand, the nanocrystalline alloy (5 h BM-SPS) shows an interesting behaviour. While the value of S increases considerably with increasing temperature there is a simultaneous increment in the value of σ, though small, across the temperature range studied.

The as cast sample exhibits a room temperature Seebeck coefficient of 52 µV/K, which increases to 64 µV/K at 373 K. Further measurement was not carried out to avoid melting of Sn-rich phase. 1 h BM-SPS sample exhibits a Seebeck coefficient of 60 µV/K at room temperature, which increases to 120 µV/K at 860 K. On the other hand in the 5 h BM-SPS sample, the Seebeck coefficient increases from 37 µV/K at room temperature to 72 µV/K at 860 K.

The as cast sample exhibits room temperature electrical conductivity of 0.8 × 10^5^ Sm^−1^ which drops to 0.73 × 10^5^ Sm^−1^ at 373 K. The 1 h BM-SPS sample exhibited a room temperature electrical conductivity of 0.89 × 10^5^ Sm^−1^ which decreases with increasing temperature to 0.71 × 10^5^ Sm^−1^ at 860 K. 5 h BM-SPS sample exhibited an electrical conductivity of 1.08 × 10^5^ Sm^−1^ at room temperature. The product S^2^σ (power factor) as a function of temperature is shown in Fig. 4c. The power factor increases gradually with temperature for both the samples. The power factor values are similar for the as-cast and 1 h BM-SPS samples. However, the 1 h BM-SPS pellet shows higher power factor than the 5 h BM-SPS samples at all temperatures studied. The 1 h BM-SPS pellet exhibited a S^2^σ value of 1.02 mWm^−1^K^−2^ at 860 K.

**Figure 5:**
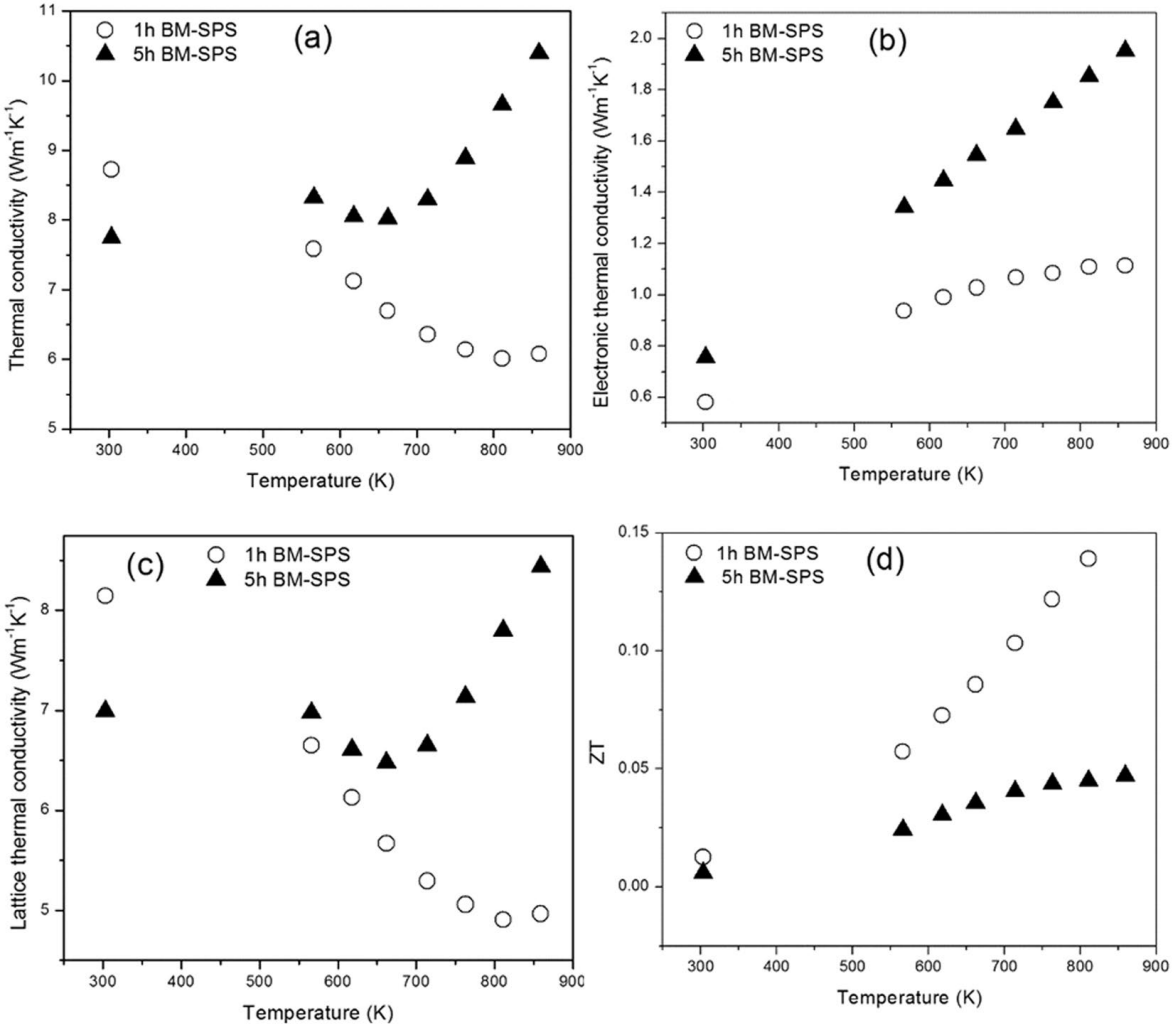
(**a**) Total thermal conductivity, (**b**) electronic thermal conductivity, (**d**) lattice thermal conductivity and (**d**) thermoelectric figure of merit (ZT) ofTi_2_NiCoSnSb alloy.

The plot of total thermal conductivity as a function of temperature is shown in Fig. 5a. The microcrystalline alloy (1 h BM-SPS) exhibited a decrease in thermal conductivity with increasing temperature whereas, the nanocrystalline alloy (5 h BM-SPS) shows an increase in thermal conductivity with increasing temperature. The electronic thermal conductivity (κ_e_) is expressed by the relation:

$${\kappa }_{e}=L\,\sigma \,T$$

where, L is the Lorenz number, σ is the electrical conductivity and T is the temperature in Kelvin scale. The Lorenz number as a function of temperature is calculated using single parabolic model approach with the scattering mode as acoustic phonon^27^. The plot of κ_e_ as a function of temperature is displayed in Fig. 5b. The κ_e_ value is higher for the 5 h BM-SPS sample across the temperature range studied. Figure 5c shows the lattice thermal conductivity (κ_l_) as a function of temperature. The room temperature lattice thermal conductivity is slightly higher for the 1 h BM-SPS sample. However, with increasing temperature, there is a continuous drop in the κ_l_ value for the 1 h BM-SPS pellet. On the other hand for the 5 h BM-SPS sample, there is a slight decrease followed by an increase in the κ_l_ value.

The room temperature dimensionless figure of merit (ZT) is almost similar for both the samples. However, with increasing milling time there is a decrease in power factor and an increase in the thermal conductivity value. This leads to the 5 h BM-SPS sample exhibiting a lower ZT across the entire temperature range studied as shown in Fig. 5d. The 1 h BM-SPS pellet exhibits a ZT of 0.144 at 860 K.

## Discussion

TiNiSn and TiCoSb are intermetallic compounds crystallizing in F-43m symmetry. The alloy Ti_2_NiCoSnSb is formed by the combination of TiNiSn and TiCoSb. The empirical rules for the phase formation in HEAs are shown in Table S3. It is evident from Table S3 that the alloy prefers to form a solid solution. Since the present alloy is a combination of two isomorphous intermetallic compounds, this can as well be termed as an extended solid solution.

Gürth *et al*. have demonstrated that TiNiSn exists as a point in an isothermal section of the Ti-Ni-Sn ternary phase diagram. It essentially means that a slight shift in composition from the centroid of the isothermal section would result in the generation of secondary phases^28^. However, even with Sn and Ti segregation present in the material, one can see the single HH phase formation in the present case without the presence of any other secondary intermetallic phases. This indicates that Ti_2_NiCoSnSb alloy has a wider HH phase field and can be treated as an extended solid solution due to high entropy effect^16^.

Additionally, the single phase formation in this alloy can also be understood by looking into the binary phase diagrams. There are 10 possible binary phase diagrams in this quinary system. Out of these Ti-Ni, Ti-Sn and Ti-Sb and Co-Sn exhibit a line compound at the equiatomic composition. Ti-Co, Ni-Sb, Co-Sb and Sn-Sb however, exhibit an extended phase field near the equiatomic composition. Ni-Co exhibits complete solubility whilst Ni-Sn shows some solubility slightly away from the equiatomic composition and forms a compound Ni_3_Sn_4_ with an extended phase field^29^. The presence of six binary systems having extended solid solution at equiatomic composition can be one of the reasons for the extended solid solubility in the present quinary alloy. In addition, MA/BM is expected to widen the phase fields of intermetallic compounds due to nanocrystallization. TiNiSn and TiCoSb ternary systems have at least one of the three possible binaries having a line compound. Hence, a slight shift in composition from the equiatomic composition can lead to the formation of secondary phases as has been reported earlier^28^. However, the process of MA has shown to accommodate slight variation in composition as reported earlier during the synthesis of TiNiSn^9^.

Although, the empirical rules may indicate the formation of a solid solution, the high enthalpy of mixing (−12 kJ/mol) as shown in Table S3, may indicate the formation of intermetallic compounds due to strong pairwise interactions. This can be an indirect justification for the formation of secondary phases in the system. Vegard’s law indicates a linear variation in lattice parameter between the two elements or compounds^30^ with variation in composition. In the present as-cast alloy, the HH phase has a lattice parameter of 0.5915 ± 0.0022 nm, which is larger than TiCoSb (0.5882 nm)^31^ and smaller than TiNiSn (0.5921 nm)^32^. Upon milling, Sn gets dissolved into the alloy and thus there is a slight increase in lattice parameter with increasing milling time.

The cast alloy has HH phase with a minor undissolved Sn, as observed from XRD analysis. The presence of Ti-rich region is not observed in the XRD pattern, possibly due to its low volume fraction. In the present system, the melting point of the elements decrease in the order of Ti > Co > Ni > Sb > Sn. As the liquid cools, the Ti-rich region is the first to solidify as primary dendrites as observed in Fig. S1. The Sn, having the lowest melting point, is the last to solidify and thus forms part of the interdendritic region. The elemental maps in Fig. 1c and point EDS analysis in Table S1 confirm the chemical composition of the three regions. The possibility of any segregation in the matrix phase was also ruled out by the elemental mapping from the matrix region as shown in Fig. S1. However, it has been observed before that intermetallic phases can have small clusters, which may not be detected by SEM analysis owing to their small size and limited fraction^33^. In order to rule out the presence of any such clusters in the HH phase, APT was carried out from a region containing HH phase.

Ball milling for 5 h gave rise to single phase HH alloy having a lattice parameter of 0.5921 ± 0.0009 nm. However, after the alloy was subjected to SPS after 1 h and 5 h ball milling, the formation of TiC is noticed, which is seen as black region in SEM. In addition, a white Ni-Sn rich phase was also observed in the SEM study, which was not observed in the XRD studies. The chemical composition of the white and black regions was identified by point EDS and is shown in Table S2. Carbon pick up during wet milling from toluene and its subsequent increase during SPS due to the graphite dies is well documented^34,35^. The Ni-Sn rich phase can possibly be Ni_3_Sn_4_ phase, which is the most readily formed phase in Ni-Sn system. Ni_3_Sn_4_ phase has been reported to form during MA stage and the retention of it is seen even after powder consolidation using SPS^9,36^. In the present study, owing to the formation of TiC, adequate concentration of Ti is not available to form the HH phase. Hence, white region corresponding to Ni-Sn rich phase gets rejected and is precipitated out. The above phenomenon can be understood better in the 5 h BM-SPS sample. The XRD pattern in Fig. 2b and the micrographs in Fig. S4 clearly show the presence of TiC (black) region. It is quite clear that the extent of TiC (black region) formation is higher in the 5 h BM-SPS sample in comparison to the 1 h BM-SPS sample. This leads to depletion of Ti from HH phase, making it destabilized thus rejecting Ni and Sn, which from Ni_3_Sn_4_ in the vicinity of Ti-rich regions. It is important to note that Ni-Sn rich regions are always found adjacent to Ti-rich regions.

**Figure 6:**
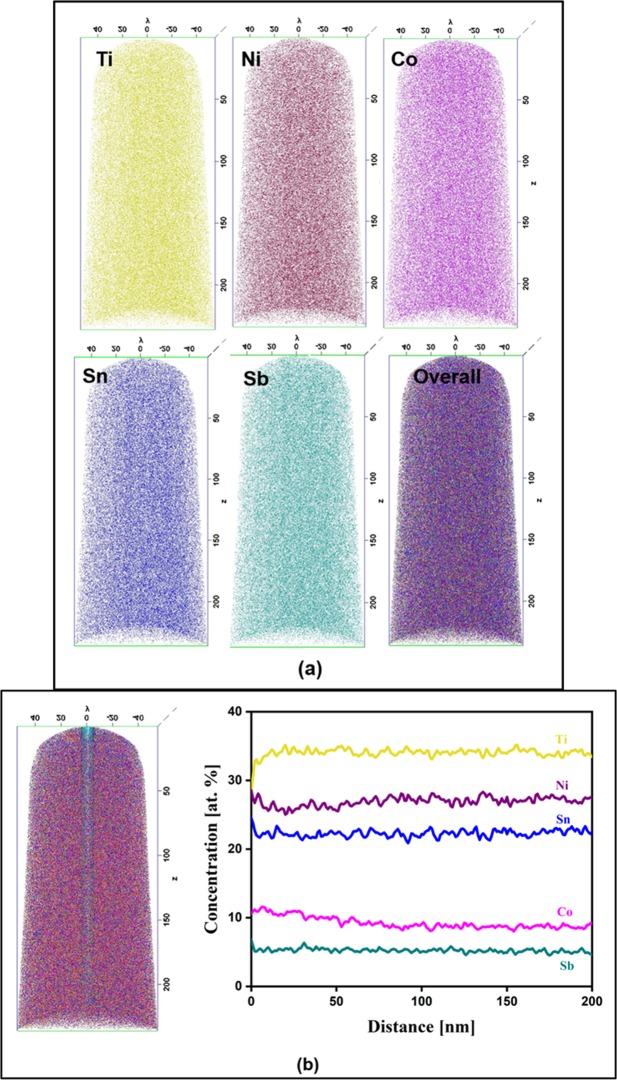
APT study on the as-cast alloy showing (**a**) elemental mapping to be devoid of segregation, (**b**) composition profile showing uniform composition across the cylinder.

**Figure 7:**
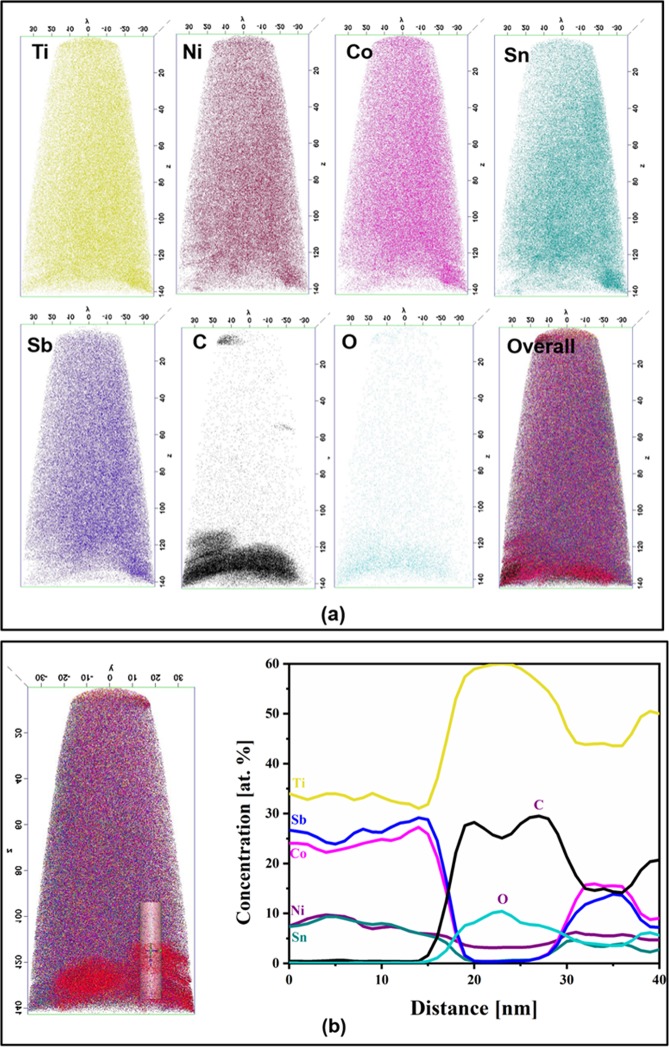
APT study on the 5 h BM-SPS alloy showing (**a**) elemental mapping to be devoid of segregation, (**b**) composition profile showing uniform composition across the cylinder.

The distribution of Ti, Ni, Co, Sn and Sb and presence of local segregation were analyzed by APT in both the alloys. The APT 3-D reconstructions of as cast alloy from the grey region that corresponds to the HH phase is shown in Fig. 6a, which indicates uniform distribution of the elements in the sample volume without any segregation. The concentration profiles were measured from the cylindrical region with the dimension 10 × 10 × 60 nm^3^ as shown in Fig. 6b. The absence of segregation in the cast alloy was further confirmed from the compositional profiles of various elements. In order to confirm the presence of Ti/O/C rich phase, APT analysis was carried out on the 5 h BM-SPS alloy. The APT 3D reconstruction of this sample from the grey and dark region are shown in Fig. 7a. The presence of O and C segregation was observed as seen in Fig. 7a,b. The 1D concentration profiles shown in Fig. 7b, indicate segregation of Ti, O and C in the region studied.

The absolute value of the Seebeck coefficient was found to increase with increasing temperature in both the alloys. The alloy exhibited n-type semiconducting behaviour and thus electrons are the major charge carriers in the sample. With increasing temperature, the thermopower almost reached a saturation in the 5 h BM-SPS sample. This can be attributed to band filling by movement of electrons from the valence band to the conduction band. The 5 h BM-SPS samples had lower absolute Seebeck coefficient values in comparison to the as cast and the 1 h BM-SPS sample and it can be attributed to the presence of larger volume fraction of TiC in the 5 h BM-SPS sample as shown in Table 1. Zou *et al*.^10^ have reported that ultrafine grained TiNiSn exhibits lower Seebeck coefficient as compared to bulk alloys studied by Kim *et al*. across all the temperature of study^10,37^. In the present study, because of the presence of secondary phases, the value of S for both the BM samples is smaller in comparison to literature reports on single phase HH alloys^7,38^. The 5 h BM-SPS sample has higher volume fraction of TiC as observed from XRD and SEM (Figs 2, S4 and Table 1) and has higher electrical conductivity in comparison to the 1 h BM-SPS sample. As discussed in the previous section, due to the formation of TiC, Ni_3_Sn_4_ forms in the alloy due to the depletion of Ti from HH phase and the resultant destabilization. Ni_3_Sn_4_ has been reported to be a metallic phase that leads to significant increase in the electrical conductivity in Zr_9_Ni_7_Sn_8_ nanocomposite^39^. This also makes the electrical conductivity of the 5 h BM-SPS sample to be higher than TiCoSb by an order of magnitude. An important point to note here is that there is not much change between the S, σ and power factor values of the as cast and 1 h BM samples. This can be attributed to the similar concentration of secondary phases as observed in Table 1.

The 5 h BM-SPS sample exhibits a simultaneous increase in S and σ. This is contrary to what is generally observed for semiconductors. This can be effected by the introduction of increased number of interfaces in the samples. It has been observed that when the dimensions of the interfaces in the sample vary in length scale from μm to nm, preferential scattering of majority carriers can occur at the expense of minority carriers^40^. It is well known that nanocrystalline samples have larger volume fraction of interfaces that lead to scattering of phonons. In the present alloy, there are three phases, namely, HH, Ni-Sn and Ti-C-O phases. The size of these regions are ultrafine in nature (varying from ~15 nm–2 μm) as shown in Figs 3 and S4 and can exhibit interfacial scattering. Such phenomena have been reported in nanocrystalline samples before^40^. However, due to higher volume fraction of TiC in the 5 h BM-SPS sample, overall improvement in power factor value is not observed. At present, the HE effect on the thermoelectric properties is still in the nascent stage of understanding and is a subject for further study. Hence the HE effect has not been elaborately discussed in the present manuscript. However, the effect of increased number of elements leading to increased entropy of mixing brings in some interesting effects in thermoelectric properties upon comparison with the two ternary systems-TiNiSn and TiCoSb. The power factor and ZT values across all temperature of measurement lie between that obtained for pure TiNiSn^7^ and TiCoSb^41^ as shown in Figs S5c and S6d, respectively. Increased number of elements in the lattice can lead to increased randomness which in turn can lead to reduced thermal conductivity. This argument holds true if we compare the κ of the HH alloy in comparison to TiCoSb. Large reduction in thermal conductivity is observed across entire temperature range of measurement as shown in Fig. S6a. Additionally at T > 800 K, κ of the 1 h BM-SPS HH alloy is nearly equal to that of TiNiSn.

TiNiSn and TiCoSb exhibit Umklapp type of scattering wherein there is continuous decrease in thermal conductivity with increasing temperature. The 1 h BM-SPS sample exhibit similar trend. However, the 5 h BM-SPS sample exhibited an increase in the total thermal conductivity. As discussed previously, the 5 h BM-SPS sample has a higher volume fraction of TiC and Ni_3_Sn_4_ phases. TiC and Ni_3_Sn_4_ phases are reported to exhibit high thermal conductivity. Additionally, TiC is known to exhibit an increase in thermal conductivity with increasing temperature^42^. There is a significant reduction in the thermal conductivity of both the samples in comparison to TiCoSb which exhibits a room temperature thermal conductivity of 24 Wm^−1^K^−1 ^^41^. The major contribution to the total thermal conductivity is from the lattice thermal conductivity as is commonly seen with HH alloys. The electronic conductivity of the 5 h BM-SPS sample is higher and this trend is also reflected in the electronic thermal conductivity of the samples. The ZT of the 5 h BM-SPS is low owing to the combined effects of low power factor and high thermal conductivity. The 1 h BM-SPS sample owing to the presence of lower amount of secondary phases gives a higher power factor and lower thermal conductivity. This leads to the 1 h BM-SPS sample having a higher ZT value of 0.144 in comparison to the 5 h BM-SPS (ZT = 0.047) sample. Although the ZT values of the samples are lower than that of TiNiSn, the values of the samples are however, higher than that of TiCoSb^7,38^.

## Conclusions

The present work reports the synthesis of a new HH type HEA for TE applications. The high entropy effect due to the addition of a large number of elements can help in evading larger hours of annealing to attain single phase. The LEAP study carried out on the HH phase confirmed complete solid solubility in the alloy. The 1 h BM-SPS sample exhibited a ZT value of 0.144 at 860 K. Due to the larger volume fraction of TiC in the 5 h BM-SPS sample, it exhibited a low ZT of 0.047 at 860 K. An interesting observation in the present study is that nanostructuring as well as secondary phase inclusions can lead to the desirable behaviour of simultaneous increase in S and σ with increasing temperature. This opens the possibility of achieving higher power factor values.

## Methods

Ti_2_NiCoSnSb alloy was prepared by VAM using elements of 99.5% purity. 3% excess Sb was added to take care of its loss due to its high vapour pressure. Ti was used as getter material to avoid oxidation. The alloy was remelted 4 times to ensure homogeneity in the sample. The samples were subsequently hand crushed into powder form using a mortar and pestle. The crushed alloys were ball milled in Fritsch Pulverisette P-5 high energy planetary ball mill in tungsten carbide (WC) vials with 10 mm WC balls. The ball to powder weight ratio was kept as 10:1. Toluene was used as process control agent (PCA) and speed of milling was 300 rpm. The as-cast alloys were ball milled for 1 h to obtain microcrystalline alloys and for 5 h to obtain nanocrystalline alloys. The milled powders were consolidated by SPS using Dr. Sinter SPS-650 of Sumitomo Metals, Japan. Sintering for all the samples was carried out at 1373 K with a dwell time of 5 min with an applied pressure of 50 MPa at a heating rate of 100 K/min to generate samples of 20 mm diameter.

The alloy was characterized by X-ray diffraction (XRD) using X’Pert Pro PanAlytical unit with Cu-Kα radiation. The single peak profile analysis using pseudo-Voigt function was used for crystallite size analysis from XRD patterns. Rietveld refinement was carried out using FULLPROF software. The thermal stability of the phases in the sintered samples were studied using a SETARAM LabSys evo differential thermal analyzer (DTA). The compositions of the alloys were estimated by energy dispersive X-ray spectroscopy (EDS) unit attached to FEI Helios G4 UX Dual Beam scanning electron microscope (SEM). The transmission electron microscopy (TEM) analysis was carried out using FEI Titan G2 60–300 high resolution TEM. The Seebeck coefficient (S) and electrical resistivity (ρ) were measured by the differential method and four probe method, respectively, using Linseis LSR-3 from room temperature to 873 K using 10 mm × 2 mm × 2 mm samples. Thermal diffusivity measurements were carried out using laser flash technique in a Netzsch LFA-427. The needle shaped APT specimens were annular milled using dual beam FEI Helios G4 UX focused ion beam/scanning electron microscopy (FIB/SEM). Atom probe characterization were performed by CAMECA LEAP 5000 XR using laser mode with a pulse frequency of 250 kHz, a focus laser energy of 30 pJ. These conditions led to individual LEAP datasets with up to 40 million atoms. The data analysis was performed with the Integrated Visualization and Analysis Software (IVAS 3.6.12) of CAMECA Instruments Inc. The Carbon analysis was carried out using G4 Icarus Series 2.

## Supplementary information

Ti2NiCoSnSb - a new half-Heusler type high-entropy alloy showing simultaneous increase in Seebeck coefficient and electrical conductivity for thermoelectric applications
