# Supplementary material for: Ti2NiCoSnSb - a new half-Heusler type high-entropy alloy showing simultaneous increase in Seebeck coefficient and electrical conductivity for thermoelectric applications
Source: Sci Rep. 2019 Mar 29;9:5331. doi: 10.1038/s41598-019-41818-6 (PMC6441071; doi:10.1038/s41598-019-41818-6)
Supplement: Supplementary file 1 — Ti2NiCoSnSb - a new half-Heusler type high-entropy alloy showing simultaneous increase in Seebeck coefficient and electrical conductivity for thermoelectric applications [file 41598_2019_41818_MOESM1_ESM.pdf]

$Ti_2NiCoSnSb$ - a new half-Heusler type high-entropy alloy showing simultaneous increase in Seebeck coefficient and electrical conductivity for thermoelectric applications

Anirudha Karati[1], M. Nagini[2], Sanyukta Ghosh[3], Rajashekhara Shabadi[4], K.G. Pradeep[2], Ramesh Chandra Mallik[3], B.S. Murty[2a], U.V. Varadaraju[1]

[1]Department of Chemistry, [2]Department of Metallurgical and Materials Engineering, Indian Institute of Technology Madras, Chennai, India

[3]Thermoelectric Materials and Devices Laboratory, Department of Physics, Indian Institute of Science, Bangalore, India

[4]Faculty of Science and Technology, UMET, University of Lille, Villeneuve-d'Ascq, France

Supplementary information

Table S1: The composition of as cast alloy and that of individual regions (N = 3) obtained from EDS analysis.

	**Overall**		**Black region**	**White region**	**Grey region**
	Nominal	Actual			
Ti	33.3	36.9±0.4	83.4±2.4	0	33.3±1.6
Ni	16.6	16.2±0.1	2.6±0.3	0	19.6±0.3
Co	16.6	17.4±0.0	3.2±1.1	0	16.2±0.5
Sn	16.6	13.8±0.2	7.7±0.6	100	14.4±0.7
Sb	16.6	15.7±0.2	3.1±0.3	0	16.4±0.1

Table S2: Composition of individual regions (N = 3) in the 1h and 5h BM-SPS alloys obtained from EDS analysis.

Element	1h BM-SPS				5h BM-SPS			
	Overall	Black Region	White region	Grey region	Overall	Black region	White region	Grey region
Ti	35.7±0.2	54.1±6.3	17.1±1.4	36.1±2.1	34.3±0.2	35.2±0.6	12.8±2.1	32.2±0.7
Ni	15.6±0.3	1.0±0.6	35.7±0.7	14.7±1.2	15.9±0.1	8.9±0.4	38.4±0.9	14.5±0.8
Co	16.9±0.3	1.0±0.9	2.8±0.4	17.1±0.4	15.5±0.5	7.6±0.2	7.5±1.0	22.5±0.6
Sn	14.2±0.4	1.7±0.7	42.8±0.5	13.7±0.3	17.1±0.9	7.2±0.2	35.4±0.7	10.9±0.7
Sb	17.6±0.3	1.8±1.1	1.6±0.5	18.4±0.3	17.2±0.2	7.2±0.1	6.0±1.3	19.9±0.5
C	-	4.6±0.6	-	-	-	24.0±0.9	-	-
O	-	39.5±2.9	-	-	-	9.9±0.7	-	-

Table S3: Phase formation criteria evaluated for $Ti_2NiCoSnSb$ alloy based on the literature.

**Parameter**	**Value**	**Prediction**
Entropy of mixing (ΔS_{mix}, R=8.314 J/mol K)	1.56 R	Solid solution
Enthalpy of mixing (ΔH_{mix}, kJ/mol)	-26.0	Intermetallic
Atomic size difference (δ, %)	4.02	Solid solution
Valence electron configuration (VEC)	6.01	Solid solution
Electronegativity ($\Delta\chi_{Pauling}$)	4.03	Intermetallic
Thermodynamic parameter (Ω)	0.75	Intermetallic

Table S4: Results of the structure refinement from X-ray powder data of HH phase synthesized by VAM and VAM + BM.

Processing route	VAM	VAM + BM
Ball Milling time (h)	-	5
R_p	4.61	5.98
R_{wp}	6.62	7.60
R_{exp}	3.59	5.94
χ^2	3.40	1.64
Lattice parameter (nm)	0.59153	0.59245
Volume (nm^3)	0.2070	0.2080
Total number of parameters used for structural refinement	29	20

R_p = profile R-factor, R_{wp} = weighted profile R-factor, R_{exp} = expected R-factor, χ^2 = Goodness of fit

Table S5: Enthalpy of formation of possible binary alloys.

Binary alloy system	Heat of formation (ΔH_f) ($kJ\text{-}mol^{-1}$)
Ti-Ni	-35
Ti-Co	-28
Ti-Sn	-21
Ti-Sb	-33
Ni-Co	0
Ni-Sn	-4
Ni-Sb	-1
Co-Sn	0
Co-Sb	2
Sn-Sb	-1
Average	-12.1

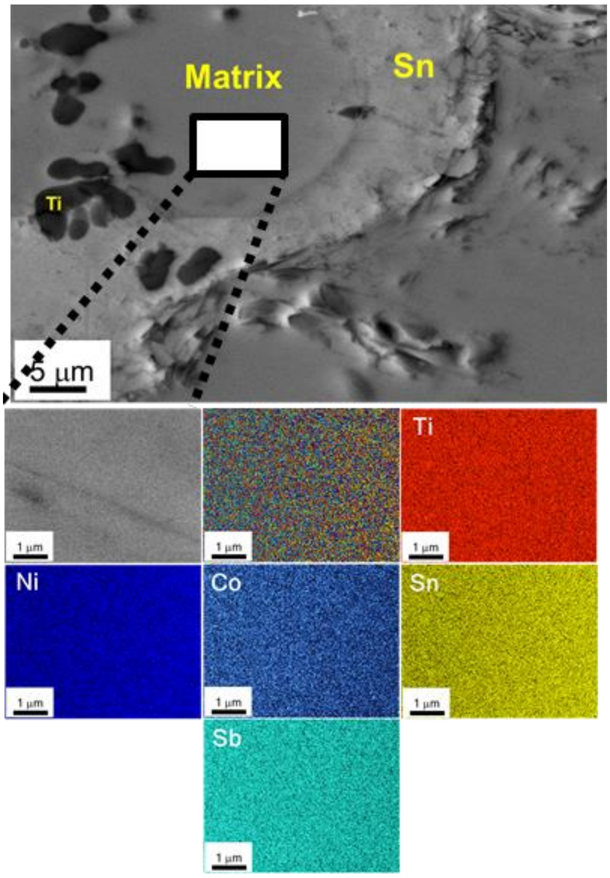

Figure S1: Elemental mapping of the as cast alloy showing absence of segregation in the matrix phase.

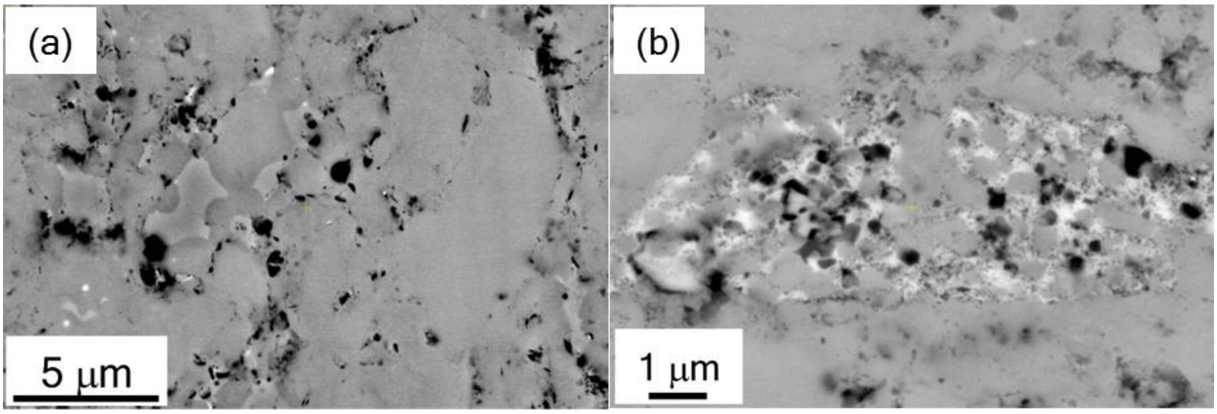

Figure S2: SEM BSE micrographs of the 1h BM-SPS alloy showing 3 phases at (a) low magnification and (b) high magnification.

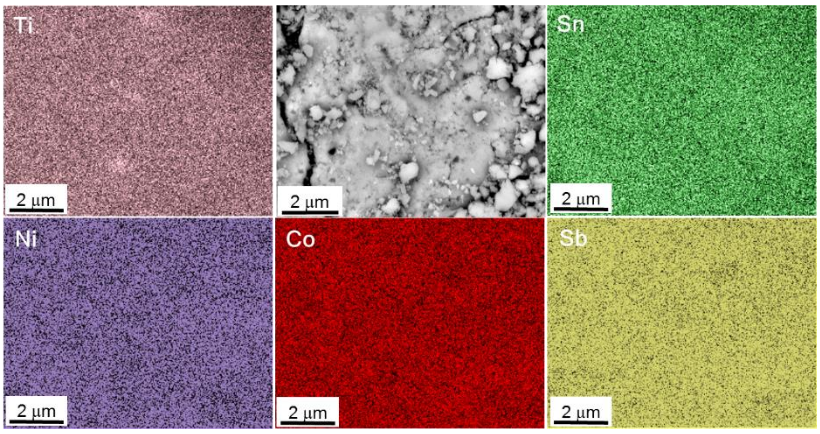

Figure S3: Elemental mapping of 5h BM powder showing absence of Sn segregation. Minor segregation in Ti is still present.

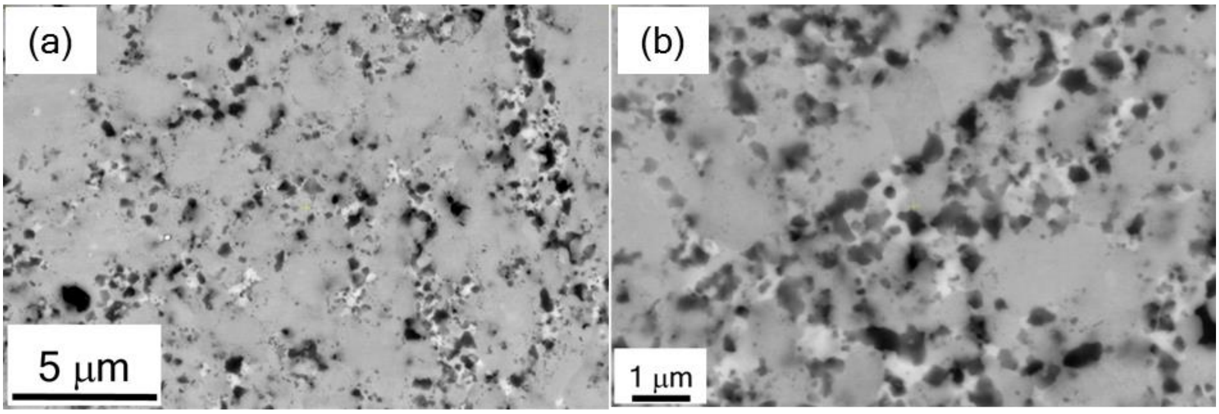

Figure S4: SEM BSE micrographs showing 3 phases at (a) low magnification and (b) high magnification in 5h BM-SPS pellet.

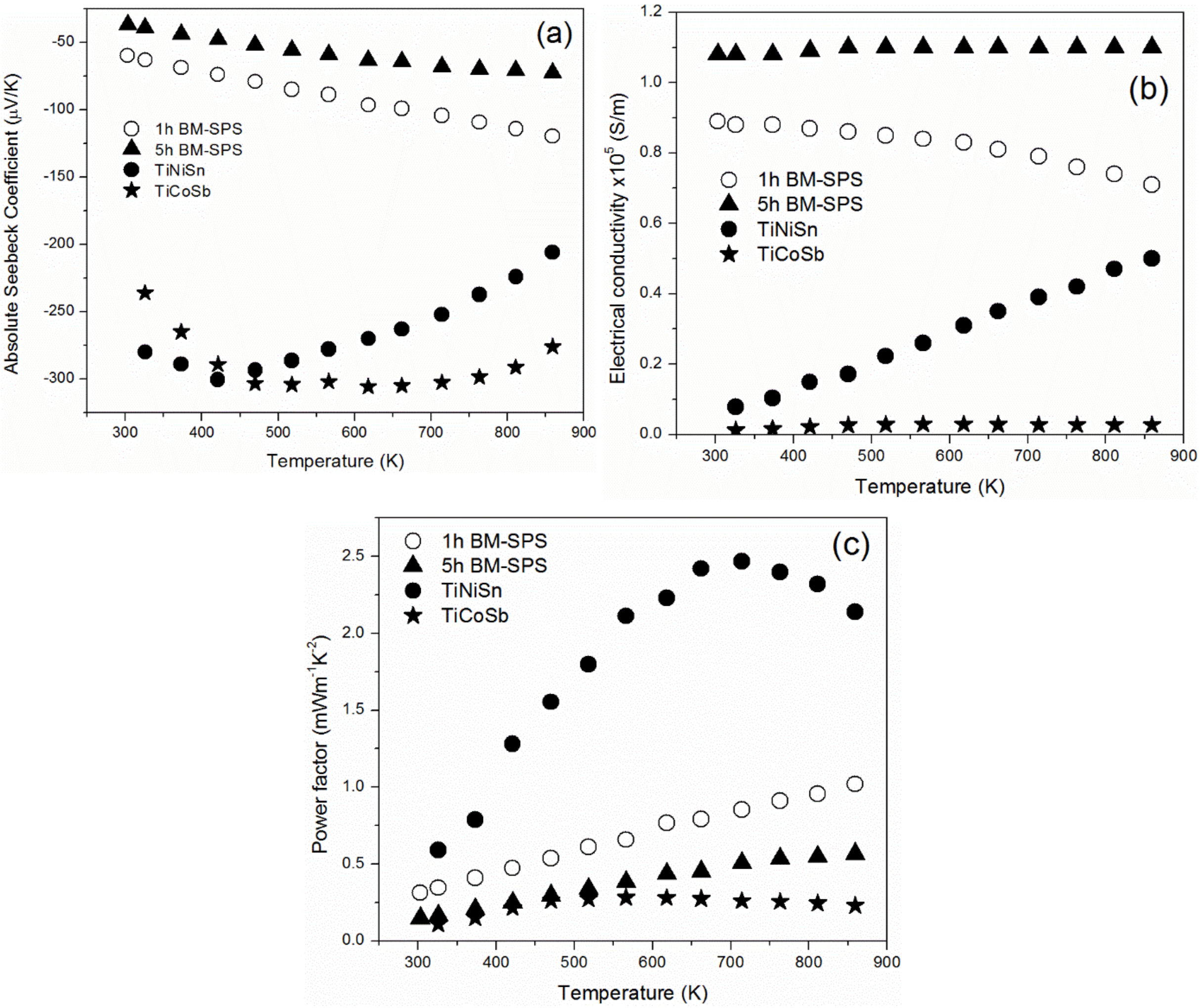

Figure S5: Seebeck coefficient, Electrical conductivity and power factor as a function of temperature for as-cast, 1h BM-SPS and 5h BM-SPS pellet.

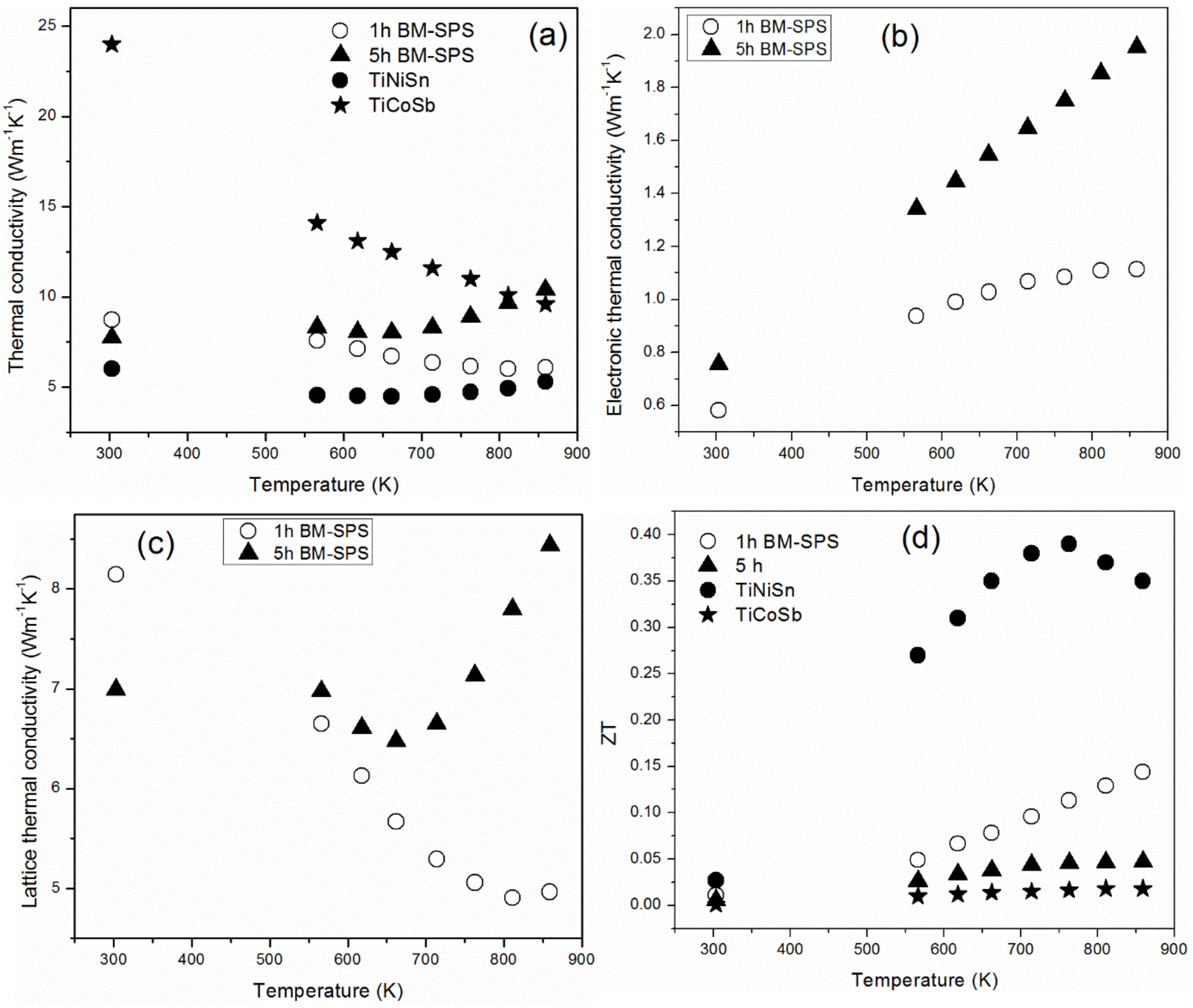

Figure S6: (a) Total thermal conductivity, (b) electronic thermal conductivity, (d) lattice thermal conductivity and (d) thermoelectric figure of merit (ZT) of$Ti_2NiCoSnSb$ alloy in comparison to TiNiSn and TioCoSb.
